# Trends in Hospital-Based Specialty Palliative Care in the United States From 2013 to 2017

**DOI:** 10.1001/jamanetworkopen.2019.17043

**Published:** 2019-12-06

**Authors:** Laura A. Schoenherr, Kara E. Bischoff, Angela K. Marks, David L. O’Riordan, Steven Z. Pantilat

**Affiliations:** 1 Division of Palliative Medicine, University of California, San Francisco

## Abstract

**Importance:**

Although palliative care (PC) historically focused on patients with cancer and those near the end of life, evidence increasingly demonstrates a benefit to patients with a broad range of serious illnesses and to those earlier in their illness. The field of PC has expanded and evolved rapidly, resulting in a need to characterize practice over time to understand whether it reflects evolving evidence and guidelines.

**Objective:**

To characterize current practice and trends among patients cared for and outcomes achieved by inpatient specialty PC services in the United States.

**Design, Setting, and Participants:**

This retrospective cohort study was performed from January 1, 2013, to December 31, 2017, at 88 US hospitals in which PC teams voluntarily participate in the Palliative Care Quality Network (PCQN), a national quality improvement collaborative. A total of 135 197 patients were referred to PCQN teams during the study period. Initial analyses of the study data were conducted from March 3 to March 21, 2018.

**Exposure:**

Inpatient PC consultation.

**Main Outcomes and Measures:**

A total of 23 standardized data elements collected by PCQN teams that provided information about the characteristics of referred patients, including age, sex, Palliative Performance Scale score, and primary diagnosis leading to PC consult; reason(s) given for the consultation; and processes of care provided by the PC team, including disciplines involved, number of family meetings held, advance care planning documentation completed, and screened for and intervened on needs.

**Results:**

A total of 135 197 patients were referred to inpatient PC (51.0% female; mean age, 71.3 years [range, 57.8-82.5 years]) and were significantly debilitated (mean Palliative Performance Scale score, 34.7%; range, 14.9%-56.8%). Cancer was the most common primary diagnosis (32.0%; range, 11.3%-93.9%), although rates decreased from 2013 to 2017 (odds ratio [OR], 0.84; 95% CI, 0.79-0.91; *P* < .001). Pain and other symptoms were common and improved significantly during the consultation period (pain: χ^2^ = 5234.4, *P* < .001; anxiety: χ^2^ = 2020.7, *P* < .001; nausea: χ^2^ = 1311.8, *P* < .001; dyspnea: χ^2^ = 1993.5, *P* < .001). Most patients were discharged alive (78.7%; range, 44.7%-99.4%), and this number increased over time (OR, 1.36; 95% CI, 1.27-1.46; *P* < .001). Compared with 2013, rates of discharge referral to clinic-based (OR, 4.00; 95% CI, 2.95-5.43; *P* < .001) and home-based PC (OR, 2.63; 95% CI, 1.92-3.61; *P* < .001) also increased significantly by 2017, whereas referrals to hospice decreased (OR, 0.56; 95% CI, 0.51-0.62; *P* < .001).

**Conclusions and Relevance:**

Inpatient PC teams cared for an increasing percentage of patients with diagnoses other than cancer and saw more patients discharged alive, consistent with guidelines recommending specialty PC for all patients with serious illness earlier in their illnesses. Most patients with symptoms improved quickly. Variation in practice and outcomes among PCQN members suggests that there are opportunities for further improvements in care.

## Key Points

**Question:**

How have the practice and outcomes of inpatient, specialty palliative care in the United States changed over time?

**Findings:**

During the 5 years examined in this cohort study of 135 197 patients, palliative care teams saw an increasing percentage of patients with diagnoses other than cancer, saw an increasing percentage of patients discharged from the hospital alive, and connected more patients with outpatient supportive services.

**Meaning:**

These trends suggest that inpatient palliative care teams are reaching a broader group of patients with serious illness and are seeing them earlier in their illnesses, consistent with emerging data and guidelines.

## Teaser

This cohort study characterizes current practice and trends among patients cared for and outcomes achieved by inpatient specialty palliative care services in the United States.

## Introduction

The field of palliative care (PC) has expanded rapidly in response to patient and practitioner demand and increasing evidence of its value in caring for the sickest and most vulnerable patients in the US health care system.^1,2,3^ The prevalence of hospital-based PC services has increased by 26% during the past decade; 67% of all hospitals and greater than 90% of hospitals with more than 300 beds currently offer PC services.^4^ Although PC historically focused on caring for people with cancer and for those near the end of life, evidence increasingly demonstrates a benefit to patients with a broad range of serious illness and to those earlier in their illnesses,^5,6,7^ and national guidelines have evolved to reflect this.^8,9,10^ In the midst of this change, there is a need to characterize PC practice to understand whether it reflects recent evidence and to guide the field to best serve people with serious illness and their families.

Published reports from single centers^11,12^ and statewide and national surveys^13,14^ provide important information about the provision of PC but a lack of standardization in data limits comparisons and the ability to understand national practice and trends. The Palliative Care Quality Network (PCQN), a multisite collaborative of PC teams from a diverse range of hospitals and health care organizations across the United States, was established to address these gaps in knowledge and to provide PC teams with patient-level outcome data to guide and improve care.^15^ We analyzed the first 5 years of the PCQN data to assess trends in the practice of hospital-based specialty PC in the United States, identify best practices, and reveal opportunities to improve care for people with serious illness.

## Methods

### Procedure

The data for this retrospective cohort study were extracted and deidentified on March 6, 2018, and include information about 135 197 patients who were referred for PC consultation by 1 of 88 PCQN member teams from January 1, 2013, to December 31, 2017. The PCQN data are collected on individual patient encounters during clinical care (on paper, electronically, or as part of routine documentation in the electronic medical record) before they are submitted to the PCQN’s secure, online database. Initial analyses of the study data were conducted from March 3 to March 21, 2018. The study was reviewed and approved by the University of California, San Francisco Institutional Review Board, which waived the need for informed consent from participants because PCQN data are deidentified and aggregated to assess quality of the care provided to patients within and between palliative care teams. This study followed the Strengthening the Reporting of Observational Studies in Epidemiology (STROBE) reporting guideline.

### The PCQN

The PCQN is a national collaborative of specialty PC teams in hospitals and health care organizations across the United States that collect standardized data on processes of care and patient-level outcomes.^15^ As of December 2017, there were 88 teams in 17 states collecting and submitting data. Although the PCQN strives for geographic diversity among its members, the network originated in California, and teams within that state continue to make up a large portion of its members. Members of the PCQN come from hospitals that vary in size (mean, 379 beds; range, 48-1126 beds) and type (66% not for profit, 19% teaching, 9% public, 1% for profit, and 4% other). This distribution is similar to national data for all hospitals (48% not for profit, 19% public, and 21% for profit), and divergences from it may be explained by the tendency for PC to be available at higher frequencies at not-for-profit and public hospitals. Moreover, hospitals in the PCQN are larger than the national average (150 beds), fitting with national trends that demonstrate a higher prevalence of PC teams at larger hospitals.^16,17^

### Data Elements

Teams in the PCQN collect a standardized set of 23 data elements for all patients seen. These data elements provide information about the characteristics of referred patients, including age, sex, Palliative Performance Scale^18^ score (a 0%-100% measure of functional status modified from the Karnofsky Performance Scale, with higher scores reflecting greater function), and primary diagnosis leading to PC consult; reason(s) given for the consultation; and processes of care provided by the PC team, including disciplines involved, number of family meetings held, advance care planning documentation completed, and screened for and intervened on needs. An issue is defined as having been intervened on if it is substantially addressed during the consultation regardless of whether it is resolved. Diagnosis categories are discrete and defined by the PCQN in a data dictionary provided to all members.^19^ Members of the PCQN document code status, discharge location, and services arranged after discharge, as well as clinical outcomes such as symptom severity. Every time patients are seen, they are asked to rate the severity of their pain, anxiety, nausea, and dyspnea at the current moment, and responses are recorded using a 4-point scale (with 0 indicating none; 1, mild; 2, moderate; and 3, severe). Teams also record whether patients are not able to self-report a response and if they were seen but not assessed for a particular symptom. Improvement is defined as a decrement of at least 1 point on the 4-point scale.

The PCQN defines a family meeting as a scheduled or spontaneous meeting between key member(s) of a PC team and a patient’s family during which a wide range of issues are discussed (eg, more than just symptoms or disposition). A PC consultation is defined as the full course of PC that a patient receives during an individual hospitalization.

### Statistical Analysis

Descriptive statistics were used to describe the distribution of measures. McNemar-Bowker tests were undertaken to examine changes in symptom scores (none, mild, moderate, or severe) between the first and second PC assessments. Binary logistic regression analyses were used to examine yearly changes (with 2013 as the referent) for categorical variables of interest: primary diagnosis (cancer vs other, cardiovascular vs other, pulmonary vs other, and neurologic vs other), discharge disposition (alive vs died in hospital), and services provided after discharge (hospice vs other, clinic-based PC vs other, and home-based PC vs other). Results of these analyses were described by adjusted odds ratios (ORs) for each subsequent year (2014-2017) compared with the reference year (2013) as well as by *P* values for overall trends across all 5 years. The ORs were adjusted to incorporate a random effect for teams to account for patient clustering. Analyses were conducted for both the complete data set and a subgroup of 40 949 patient encounters submitted by 11 teams that contributed data throughout the 5-year study period. SPSS, version 23 for Mac (SPSS Inc) was used to conduct all analyses, and 2-sided α < .05 was used to determine statistical significance.

## Results

### Patient and Consult Characteristics

**Figure 1.**
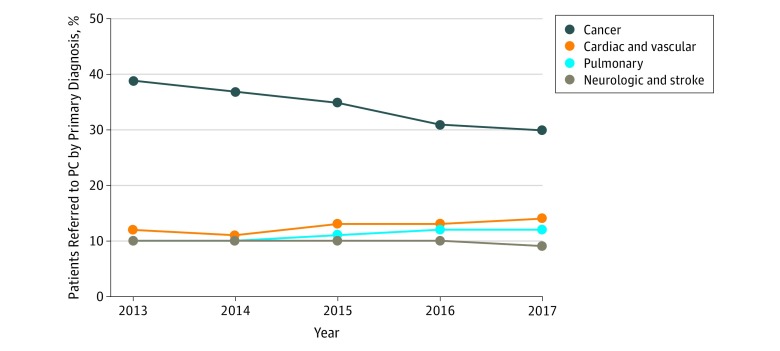
Primary Diagnosis of Patients Referred for Inpatient Palliative Care (PC) Consultation From January 1, 2013, to December 31, 2017

Patient demographics varied significantly across PC teams (Table 1). A total of 135 197 patients were referred to inpatient PC (mean age, 71.3 years; range of means among teams, 57.8-82.5 years) and were significantly debilitated (mean Palliative Performance Scale score, 34.7%; range, 14.9%-56.8%). The most common diagnoses leading to PC consultation were cancer (32.0%; range, 11.3%-93.9%), cardiovascular disease (13.2%; range, 0%-29.0%), and pulmonary disease (11.3%; range, 0%-26.0%). Between 2013 and 2017, the percentage of referrals for patients with cancer decreased (OR, 0.84; 95% CI, 0.79-0.91; *P* < .001), whereas the percentage of referrals for patients with cardiovascular disease increased (OR, 1.12; 95% CI, 1.02-1.24; *P* < .001) (Figure 1 and eTable 1 in the Supplement). Advance care planning was the most common reason for PC consultation (73.5%; range, 39.9%-97.4%) (Table 1), a finding discussed in more detail in previous work.^20^

**Table 1.** Patient, Consultation, Team, and Discharge Characteristics

Characteristic	Finding^a^
**Patient and Consult Characteristics**	
Age, mean (range), y (n = 135 093)	71.3 (57.8-82.5)
Female sex (n = 135 150)	68 890 (51.0) [43.3-72.1]
Palliative Performance Scale score, mean (range) (n = 114 508)	34.7 (14.9-56.8)
Primary diagnosis (n = 126 554)	
Cancer	40 531 (32.0) [11.3-93.9]
Cardiac or vascular	16 741 (13.2) [0-29.0]
Pulmonary	14 270 (11.3) [0-26.0]
Neurologic or stroke	12 235 (9.7) [0-16.5]
Complex chronic conditions or failure to thrive	10 927 (8.6) [0-28.3]
Infectious, immunologic, or HIV	5553 (4.4) [0-17.6]
Dementia	5358 (4.2) [0-13.3]
Hepatic	4402 (3.5) [0-12.1]
Renal	3952 (3.1) [0-7.6]
Gastrointestinal	3705 (2.9) [0-5.5]
Trauma	2776 (2.2) [0-7.0]
Hematology	1658 (1.3) [0-8.1]
Vascular	1102 (0.9) [0-2.1]
Congenital or chromosomal conditions	152 (0.1) [0-0.3]
In utero complication or condition	21 (0.0) [0-0.1]
Other	3171 (2.5) [0-48.6]
Location of care at time of consultation (n = 131 616)	
Medical or surgical floor	52 143 (39.6) [0.1-96.9]
Telemetry or step-down unit	31 266 (23.8) [0-48.9]
Critical care	30 414 (23.1) [0-59.7]
Emergency department	6603 (5.0) [0-21.0]
Other	11 190 (8.05) [0-100]
Reason for referral (n = 131 417)^b^	
Advance care planning	96 528 (73.5) [39.9-97.4]
Pain management	24 233 (18.4) [0-72.2]
Hospice referral or discussion	21 404 (16.3) [0.5-68.4]
Other symptom management	18 855 (14.3) [0-56.8]
Comfort care	10 585 (8.1) [0-58.5]
Code status at time of consult (n = 126 267)	
Full code	68 221 (54.0) [13.0-88.5]
DNR or DNI	50 472 (40.0) [2.9-84.9]
Partial code	7574 (6.0) [0-38.9]
Advance care planning documentation in medical record at time of consultation	
Advance directive (n = 128 948)	31 113 (24.1) [1.7-90.8]
POLST (n = 127 402)	15 473 (12.1) [0-46.2]
**Processes of Care**	
Team discipline involved in PC consultation (n = 120 463)	
Physician	65 192 (54.1) [0-100]
Social worker	47 505 (39.4) [0-87.0]
Registered nurse	43 564 (36.2) [0-99.7]
Nurse practitioner	35 490 (29.5) [0-97.1]
Chaplain	33 721 (28.0) [0-87.2]
Hospital length of stay before PC consultation, mean (range), d (n = 131 876)	4.8 (1.7-11.1)
Days followed by PC team, mean (range), d (n = 131 680)	5.7 (1-13.6)
No. of family meetings held per consultation, mean (range) (n = 118 970)	1.3 (0.1-3.4)
PC needs screened positive	
Advance care planning (n = 125 883)	94 179 (74.8) [0.3-98.4]
Psychosocial (n = 125 685)	61 027 (48.6) [0.5-98.5]
Other symptom management (n = 127 108)	57 974 (45.6) [0.1-87.0]
Pain management (n = 127 108)	50 994 (40.1) [0.4-73.7]
Spiritual (n = 122 711)	37 499 (30.6) [0-91.8]
PC needs intervened on^c^	
Advance care planning (n = 94 289)	87 921 (93.2) [54.5-98.6]
Psychosocial (n = 61 155)	56 147 (91.8) [30.0-98.7]
Spiritual (n = 37 633)	33 790 (89.8) [12.1-98.8]
Other symptom management (n = 58 087)	51 552 (88.7) [23.5-100]
Pain management (n = 51 107)	45 142 (88.3) [4.6-98.3]
**Discharge Characteristics**	
Alive (n = 126 944)	99 900 (78.7) [44.7-99.4]
Location^d^ (n = 94 438)	
Home	44 619 (47.2) [30.0-75.6]
Extended care facility	20 141 (21.3) [0.5-49.1]
Hospital inpatient^e^	13 554 (14.4) [0-76.7]
Long-term acute care	2687 (2.8) [0-22.7]
Other	13 437 (14.2) [1.1-49.3]
Services provided^d^	
Hospice (n = 84 594)	30 275 (35.8) [3.1-78.9]
Home nursing (n = 84 487)	13 104 (15.5) [0-46.4]
Clinic-based PC (n = 84 443)	3806 (4.5) [0-71.9]
Home-based PC (n = 84 443)	3779 (4.5) [0-45.3]
No services provided (n = 84 596)	29 849 (35.3) [0-96.0]
Code status at time of discharge^d^ (n = 49 600)	
Full code	17 744 (35.8) [7.4-100]
DNR or DNI	28 688 (57.8) [0-85.7]
Partial code	2750 (5.5) [0-44.2]
Unknown	418 (0.8) [0-18.0]
Advance care planning documentation in medical record at time of discharge^d^	
Advance directive (n = 89 700)	3134 (3.5) [0-15.4]
POLST (n = 89 668)	12 625 (14.1) [0-43.5]

Abbreviations: DNI, do not intubate; DNR, do not resuscitate; PC, palliative care; POLST, physician orders for life-sustaining treatment.

^a^ Data are presented as number (percentage) [percentage range among teams] unless otherwise indicated. Discrepancies between the listed sample size and the full study sample size of 135 197 constitute missing data and/or excluded subpopulations (eg, discharge data were collected only for patients discharged alive).

^b^ More than 1 reason could be provided.

^c^ Teams initially reported only the results of their needs assessment. Data elements for need intervention were added later, accounting for the smaller number here relative to above.

^d^ Among patients discharged alive.

^e^ Team signed off before patient was discharged.

### Team Structure and Services Provided

Practitioners from multiple disciplines were involved in PC consultations, with physicians most commonly involved (54.1% of consultations; range, 0%-100%), followed by social workers (39.4%; range, 0%-87.0%), registered nurses (36.2%; range, 0%-99.7%), nurse practitioners (29.5%; range, 0%-97.1%), and chaplains (28.0%; range, 0%-87.2%) (Table 1). Overall, 29 445 of 117 301 patients (25.1%) were seen by PC practitioners from 3 or more disciplines during their consultation.

Consultations were requested a mean of 4.8 days into the hospital stay (range, 1.7-11.1 days), and PC teams followed patients for a mean of 5.7 days (range, 1-13.6 days). Teams conducted a mean of 1.3 family meetings per consultation (median, 1.0; range, 0.1-3.4), with 25.9% of patients having none, 43.8% having 1, 18.0% having 2, and 12.2% having 3 or more.

During the consultation, PC teams screened patients for a core set of PC needs, regardless of the reason(s) for the consultation. Most patients were found to have needs pertaining to advance care planning (74.8%), and nearly half had needs relating to pain (40.1%), other symptoms (45.6%), and psychosocial concerns (48.6%). Patients were less likely to be screened for spiritual needs (30.6%). When a need was identified, PC teams reported that they intervened most of the time (93.2% of consultations for advance care planning needs, 91.8% for psychosocial needs, 89.8% for spiritual needs, 88.7% for nonpain symptoms, and 88.3% for pain).

Code status of patients at the time of consultation varied widely across teams. Code status was clarified for 54.0% of patients during the PC consultation, and substantially more patients expressed a preference for do not resuscitate and do not intubate status after being seen by PC practitioners (40.0% before consultation and 57.8% after consultation).

### Outcomes

#### Symptom Management

Teams assessed symptoms for most patients (Table 2). Of these, more than one-quarter were too ill to report a score (27.0% for pain, 28.8% for anxiety, 27.3% for dyspnea, and 27.6% for nausea). Among those patients able to rate their symptoms, pain was most prevalent, with 29.9% of assessed patients reporting moderate to severe pain at the baseline assessment by the PC team, followed by anxiety (12.9%), dyspnea (12.2%), and nausea (5.5%).

**Table 2.** Symptom Scores at First Assessment

Variable	No. (%) of Patients			
	Pain (n = 95 364)	Anxiety (n = 95 370)	Dyspnea (n = 95 502)	Nausea (n = 95 225)
Patients seen				
Team did not assess	7388 (7.7)	10 197 (10.7)	8154 (8.5)	8326 (8.7)
Patient unable to rate	25 715 (27.0)	27 465 (28.8)	26 093 (27.3)	26 279 (27.6)
Reported score				
None	30 831 (49.5)	37 291 (64.6)	42 962 (70.1)	51 765 (85.4)
Mild	12 829 (20.6)	12 979 (22.5)	10 801 (17.6)	5547 (9.2)
Moderate	11 345 (18.2)	5754 (10.0)	5402 (8.8)	2366 (3.9)
Severe	7256 (11.7)	1684 (2.9)	2090 (3.4)	941 (1.6)
Total^a^	62 261 (100)	57 708 (100)	61 255 (100)	60 619 (100)

^a^ Among patients who were able to rate their symptoms.

**Figure 2.**
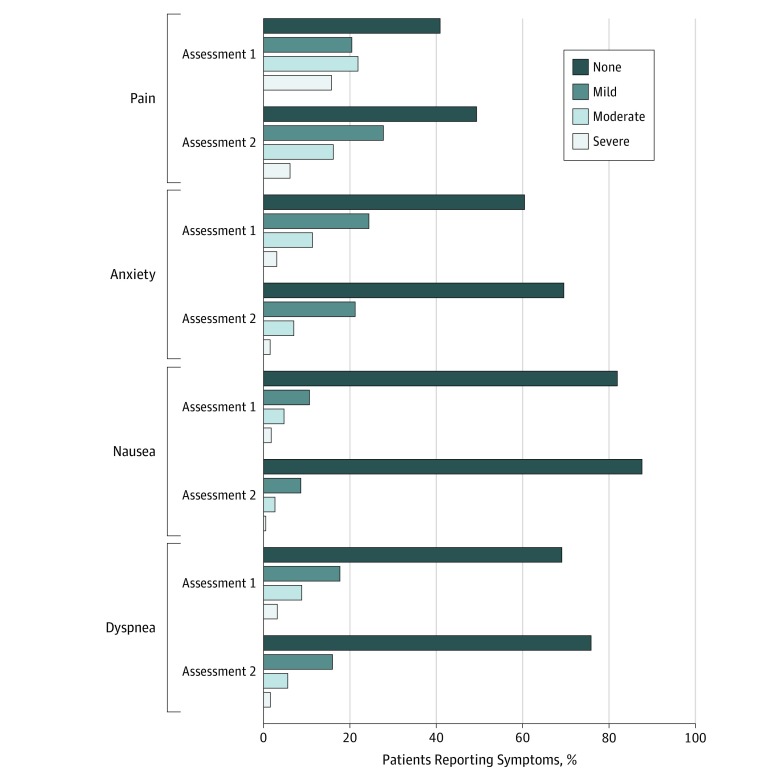
Change in Symptom Scores From First to Second Assessment Pain: χ^2^ = 5234.4, *P* < .001; anxiety: χ^2^ = 2020.7, *P* < .001; nausea: χ^2^ = 1311.8, *P* < .001; and dyspnea: χ^2^ = 1993.5, *P* < .001.

Among patients with moderate to severe symptoms at first assessment, most reported an improved score at their second assessment (pain, 69.2% [range, 40.0%-100%]; anxiety, 69.0% [range, 36.6%-94.7%]; nausea, 79.1% [range, 45.5%-91.4%]; and dyspnea, 66.8% [range, 36.4%-100%]), and overall symptom scores improved significantly for all 4 measured symptoms during the same time (pain: χ^2^ = 5234.4, *P* < .001; anxiety: χ^2^ = 2020.7, *P* < .001; nausea: χ^2^ = 1311.8, *P* < .001; dyspnea: χ^2^ = 1993.5, *P* < .001) (Figure 2).

#### Discharge Characteristics

**Figure 3.**
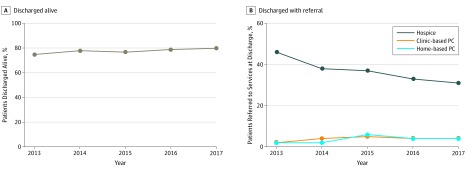
Patient Discharge A and B, Percentage of patients discharged alive (A) and with a referral to hospice, clinic-based palliative care (PC), or home-based PC services (B) from January 1, 2013, to December 31, 2017.

Most patients seen by the PC team were discharged from the hospital alive (78.7%; range, 44.7%-99.4%), and they were most commonly discharged to the home (Table 1). The percentage of patients discharged alive increased from 75% in 2013 to 80% in 2017 (OR, 1.36; 95% CI, 1.27-1.46; *P* < .001) (Figure 3A and eTable 2 in the Supplement). Rates of referral to clinic-based and home-based PC also increased during the 5 years studied, from 2% in 2013 to 4% in 2017 for clinic-based PC (OR, 4.00; 95% CI, 2.95-5.43; *P* < .001) and from 2% in 2013 to 4% in 2017 for home-based services (OR, 2.63; 95% CI, 1.92-3.61; *P* < .001) (Figure 3B and eTable 3 in the Supplement). Rates of referral to hospice decreased from 46% to 31% during the same period (OR, 0.56; 95% CI, 0.51-0.62; *P* < .001).

#### Subgroup Analysis

Trends observed within the subgroup of teams that contributed data for each of the 5 years studied were similar to those found across the entire data set. Within the subgroup, the percentage of patients with cancer decreased over time (OR, 0.83; 95% CI, 0.80-0.90; *P* < .001). Although the odds of referrals for patients with cardiovascular disease increased overall, the odds were not significantly different in 2017 compared with 2013 (OR, 1.11; 95% CI, 0.99-1.24; *P* = .06). The percentage of patients discharged alive and the percentages referred to clinic- and home-based PC increased over time (alive: OR, 1.29 [95% CI, 1.19-1.40], *P* < .001; clinic-based: OR, 3.05 [95% CI, 2.22-4.20], *P* < .001; and home-based: OR, 2.83 [95% CI, 2.05-3.93], *P* < .001), whereas the percentage of patients referred to hospice decreased (OR, 0.56; 95% CI, 0.51-0.62; *P* < .001).

## Discussion

With standardized data on more than 135 000 patients across 88 PC teams at diverse hospitals across the United States during 5 years, analysis of the PCQN data set suggests that trends in the practice of inpatient specialty PC reflect evidence and national guidelines recommending PC for all people with serious illness regardless of diagnosis and involvement of PC earlier in their illnesses. The wide variation in practice that we found identifies benchmarks and highlights opportunities to improve care by understanding better performers.

Among patients referred for PC consultation, cancer was the most common primary diagnosis, although the proportion of patients with cancer decreased significantly during the 5 years studied. In addition, most patients were discharged alive and were most likely to be discharged to their homes. Furthermore, the percentage of patients discharged alive increased over time, suggesting that PC teams may have been seeing patients earlier in their illnesses and were increasingly effective at facilitating a safe transition home. The presumption that patients were being seen earlier in their illnesses was further supported by decreased rates of hospice referral and the increased mean Palliative Performance Scale score of patients seen by PC teams during the study period. Patients were increasingly likely to be referred to outpatient PC services (home- and clinic-based programs) at the time of hospital discharge. This increased use may have been attributable to increased availability of such services that likely contributed to the safe discharge of more patients with serious illness over time. Increased referral to these community-based programs and seeing patients earlier in their illnesses may also be associated with the lower rates of hospice referral over time, although our data cannot definitively answer this question. Taken together, these findings suggest that that PC teams are reaching a broader group of patients with serious illness and seeing them earlier in their illnesses.

Our data indicate that, despite initially high rates of pain and other symptoms, patients experienced significant symptom improvement by the second PC assessment, suggesting that PC teams were able to meaningfully affect their patients’ quality of life. Deeper understanding of the structures and processes of care used by PCQN teams with better performance could elucidate factors associated with symptom improvement. Identifying these best practices, implementing them broadly to evaluate their generalizability, and disseminating the most effective strategies could result in significant improvements in the quality and consistency of care provided by PC teams nationally.

Our data revealed significant variation in all aspects of care across PCQN members. For example, some teams typically cared for patients younger than the overall average, and the percentage of patients discharged from the hospital varied more than 2-fold. Heart disease is nationally the leading cause of death and the most common reason for hospitalization among Medicare recipients, but patients with heart disease accounted for only 13% of PCQN referrals, with variation among teams ranging from 0% to 25% of consultations.^21^ To the extent that this variation reflects differences in structures and processes of care, the findings may provide insights for teams wishing to emulate these practices. Our findings also showed that although most patients were seen by a physician, fewer than half were seen by other members of the interdisciplinary team and only one-quarter were seen by practitioners from at least 3 disciplines despite national guidelines recommending interdisciplinary care as core to PC.^10^ Further analysis of PCQN data that examines associations between interdisciplinary care and outcomes could provide evidence to support this approach.

Our findings add to data published by other national and international PC registries, such as the Global Palliative Care Quality Alliance/Quality Data Collection Tool^22^ and the National Palliative Care Registry^23^ in the United States, the Danish Palliative Care Database^24^ in Denmark, the Swedish Register of Palliative Care^25^ in Sweden, and the Palliative Care Outcomes Collaboration^26^ in Australia. Although data from these registries are not directly comparable to those from the PCQN, they suggest that patients seen by PC teams in the United States are more likely to have noncancer diagnoses and are less likely to die during the consultation course than those seen by PC teams internationally.^27,28^ Harmonization of data elements across international registries could provide further insights into best practices and lead to improvements in care globally.

### Limitations

This study has limitations. The data were collected by practitioners during clinical care. As a result, not every data element was recorded for each patient, and it is possible that missing data affected our findings. Despite this, participating teams were able to collect a significant amount of standardized, meaningful data that included patient-level outcomes. Participating teams received training and used a data dictionary^19^ to standardize assessment and documentation of data. Nonetheless, some variation in processes and outcomes may have resulted from remaining differences in how practitioners interpreted data elements. Teams continued to join the PCQN throughout the study period, but trends observed across all teams did not differ meaningfully from those within the subset that contributed data across all 5 years, suggesting that the dynamic nature of the data set did not skew the study’s results.

Although PCQN teams represent hospitals of varying size and type across the United States, it is possible that teams that choose to voluntarily participate in a quality improvement collaborative differ meaningfully from those that do not, potentially limiting the generalizability of our data. In addition, although these data provide benchmarks for care provided by PC teams, they do not address how care provided by PC teams compares with care provided to seriously ill patients not seen by PC teams. Such comparisons would be helpful in understanding the role of specialty PC but are beyond the scope of the PCQN. Furthermore, patients referred for specialty PC may be at increased risk of readmission, and we had no way of determining whether individual patients were captured more than once in our data set across separate hospital admissions.

## Conclusions

The PCQN data set analyzed here provides a unique window into current practice of PC in the United States and trends in care over time, suggesting that PC in acute care hospitals is expanding to patients with diagnoses other than cancer and to those earlier in their illnesses. There has been expansion in hospital-based PC during the past decade, and national reports call for further increases in specialty PC going forward. Our findings may guide further improvements in care for people with serious illness, and analysis of better performers can help to improve care broadly.

## Notes

Supplement. **eTable 1.** Primary Diagnosis of Patients Referred for Inpatient PC Consult Between January 2013 and December 2017 **eTable 2.** Percentage of Patients Discharged Alive Between January 2013 and December 2017 **eTable 3.** Percent of Patients Discharged With a Referral to Hospice, Clinic-Based PC, or Home-Based PC Services Between January 2013 and December 2017
