# Supplementary material for: Trends in Hospital-Based Specialty Palliative Care in the United States From 2013 to 2017
Source: JAMA Netw Open. 2019 Dec 6;2(12):e1917043. doi: 10.1001/jamanetworkopen.2019.17043 (PMC6902777; doi:10.1001/jamanetworkopen.2019.17043)
Supplement: Supplement. — eTable 1. Primary Diagnosis of Patients Referred for Inpatient PC Consult Between January 2013 and December 2017 eTable 2. Percentage of Patients Discharged Alive Between January 2013 and December 2017 eTable 3. Percent of Patients Discharged With a Referral to Hospice, Clinic-Based PC, or Home-Based PC Services Between January 2013 and December 2017 [file jamanetwopen-2-e1917043-s001.pdf]

Supplementary Online Content

Schoenherr LA, Bischoff KE, Marks AK, O'Riordan DL, Pantilat SZ. Trends in hospital-based specialty palliative care in the United States from 2013 to 2017. *JAMA Netw Open*. 2019;2(12):e1917043. doi:10.1001/jamanetworkopen.2019.17043

eTable 1. Primary Diagnosis of Patients Referred for Inpatient PC Consult Between January 2013 and December 2017

eTable 2. Percentage of Patients Discharged Alive Between January 2013 and December 2017

eTable 3. Percent of Patients Discharged With a Referral to Hospice, Clinic-Based PC, or Home-Based PC Services Between January 2013 and December 2017

This supplementary material has been provided by the authors to give readers additional information about their work.

© 2019 Schoenherr LA et al. *JAMA Network Open.*

eTable 1. Primary Diagnosis of Patients Referred for Inpatient PC Consult Between January 2013 and December 2017

Primary Diagnosis	2013	2014	2015	2016	2017	p
	OR	*OR (95% CI)*	*OR (95% CI)*	*OR (95% CI)*	*OR (95% CI)*	
Cancer	1.0	1.07 (0.97, 1.16)	0.95 (0.88, 1.02)	0.91 (0.84, 0.97)	0.84 (0.79, 0.91)	<0.001
Cardiac/vascular	1.0	0.84 (0.75, 0.95)	1.03 (0.93, 1.15)	1.05 (0.95, 1.17)	1.12 (1.02, 1.24)	<0.001
Pulmonary	1.0	0.88 (0.78, 0.99)	0.98 (0.88, 1.10)	0.98 (0.87, 1.09)	0.95 (0.85, 1.06)	0.06
Neurologic/stroke	1.0	1.02 (0.91, 1.16)	1.02 (0.91, 1.14)	1.01 (0.91, 1.13)	0.97 (0.87, 1.09)	0.3

Odds Ratio (95% Confidence Interval): The odds of patients being referred to PC with a primary diagnosis (cancer vs. other; cardiac/vascular vs. other; pulmonary vs. other; neurologic/stroke vs. other) each year of data collection (2013 referent). Analyses were adjusted for variations between primary diagnosis referral among PC teams.

eTable 2. Percentage of Patients Discharged Alive Between January 2013 and December 2017

Discharge Disposition	2013	2014	2015	2016	2017	p
	OR	*OR (95% CI)*	*OR (95% CI)*	*OR (95% CI)*	*OR (95% CI)*	
Discharged Alive	1.0	1.17 (1.08, 1.27)	1.21 (1.12, 1.30)	1.30 (1.21, 1.40)	1.36 (1.27, 1.46)	<0.001

Odds Ratio (95% Confidence Interval): The odds of patients being discharged alive each year of data collection (2013 referent). Analyses were adjusted for variations between discharge disposition among PC teams.

© 2019 Schoenherr LA et al. *JAMA Network Open.*

eTable 3. Percent of Patients Discharged with a Referral to Hospice, Clinic-Based PC, or Home-Based PC Services Between January 2013 and December 2017

**Discharge Service**	**2013**	**2014**	**2015**	**2016**	**2017**	**p**
	OR	*OR (95% CI)*	*OR (95% CI)*	*OR (95% CI)*	*OR (95% CI)*	
Hospice	1.0	0.73 (0.67, 0.82)	0.63 (0.58, 0.70)	0.58 (0.53, 0.63)	0.56 (0.51, 0.62)	<0.001
Clinic–Based PC	1.0	1.99 (1.43,2.76)	3.32 (2.44, 4.52)	3.64 (2.68, 4.94)	4.00 (2.95, 5.43)	<0.001
Home-based PC	1.0	0.88 (0.61, 1.27)	2.74 (1.99, 3.77)	2.52 (1.84, 3.46)	2.63 (1.92, 3.61)	<0.001

Odds Ratio (95% Confidence Interval): The odds of patients being discharged with services (hospice vs. other; clinic-based PC vs. other; home-based PC vs. other) each year of data collection (2013 referent). Analyses were adjusted for variations between discharge referral location among PC teams.

© 2019 Schoenherr LA et al. *JAMA Network Open.*
